# NMR Studies on Structure and Dynamics of the Monomeric Derivative of BS-RNase: New Insights for 3D Domain Swapping

**DOI:** 10.1371/journal.pone.0029076

**Published:** 2012-01-12

**Authors:** Roberta Spadaccini, Carmine Ercole, Maria A. Gentile, Domenico Sanfelice, Rolf Boelens, Rainer Wechselberger, Gyula Batta, Andrea Bernini, Neri Niccolai, Delia Picone

**Affiliations:** 1 Dipartimento di Scienze Biologiche ed Ambientali, Università del Sannio, Benevento, Italy; 2 Dipartimento di Chimica, Università degli Studi di Napoli “Federico II”, Napoli, Italy; 3 Department of NMR Spectroscopy, Bijvoet Center for Biomolecular Research, Utrecht University, Utrecht, The Netherlands; 4 Institute of Chemistry, University of Debrecen, Debrecen, Hungary; 5 Dipartimento di Biotecnologie, Università degli Studi di Siena, Siena, Italy; Università di Napoli Federico II, Italy

## Abstract

Three-dimensional domain swapping is a common phenomenon in pancreatic-like ribonucleases. In the aggregated state, these proteins acquire new biological functions, including selective cytotoxicity against tumour cells. RNase A is able to dislocate both N- and C-termini, but usually this process requires denaturing conditions. In contrast, bovine seminal ribonuclease (BS-RNase), which is a homo-dimeric protein sharing 80% of sequence identity with RNase A, occurs natively as a mixture of swapped and unswapped isoforms. The presence of two disulfides bridging the subunits, indeed, ensures a dimeric structure also to the unswapped molecule. *In vitro*, the two BS-RNase isoforms interconvert under physiological conditions. Since the tendency to swap is often related to the instability of the monomeric proteins, in these paper we have analysed in detail the stability in solution of the monomeric derivative of BS-RNase (mBS) by a combination of NMR studies and Molecular Dynamics Simulations. The refinement of NMR structure and relaxation data indicate a close similarity with RNase A, without any evidence of aggregation or partial opening. The high compactness of mBS structure is confirmed also by H/D exchange, urea denaturation, and TEMPOL mapping of the protein surface. The present extensive structural and dynamic investigation of (monomeric) mBS did not show any experimental evidence that could explain the known differences in swapping between BS-RNase and RNase A. Hence, we conclude that the swapping in BS-RNase must be influenced by the distinct features of the dimers, suggesting a prominent role for the interchain disulfide bridges.

## Introduction

Protein subunits may interchange part of their polypeptide chain, giving rise to inter-twinned dimers, or even higher order aggregated forms. This phenomenon, also known as 3D domain swapping, is often accompanied by additional biological activities with respect to the parent monomeric protein, leading to new functions or even to toxic effects associated to deposition diseases [1]. To date, neither the molecular mechanism of 3D domain swapping or its predictability have been elucidated (for a recent review, see [2]).

For historical reasons [3], but mainly for the variety of oligomers formed, bovine pancreatic ribonuclease (the well-known RNase A) is considered a prototype of 3D domain swapped proteins: dimers, trimers, tetramers or even higher order oligomers of this protein have been extensively characterized, from structural and functional point of view [4]. This protein provided also the first experimental evidence that a single polypeptide chain may dislocate both N- and C-termini [5], [6], even in the same oligomeric structure [7]. The capability to dislocate N-termini is a feature of other mammalian ribonucleases [8], [9], which often in the aggregated state acquire a selective cytotoxicity towards tumour cells [10]. However, all the multimeric forms of RNase A and RNase-like proteins for biophysical studies have been prepared in vitro, under non-native conditions produced by modifying either the environmental physico-chemical conditions [11], or the protein sequence [12], [13]. Recently RNase A has been shown to form a minor population of dimers when it is produced in bovine cells [14]. However, only BS-RNase, which shares 80% sequence identity with RNase A and a common catalytic mechanism [15], offers the possibility to study the swapping process *in vitro* under mild physiological conditions. The swapping endows BS-RNase with new biological properties, including selective cytotoxicity towards tumour cells [10]. In contrast to RNase A and all other pancreatic-like ribonucleases, which are monomeric in their native state, BS-RNase is a dimer constituted by two identical subunits linked through two disulfide bridges, as well as by non-covalent interactions. The native enzyme is isolated from seminal vesicles or bull semen [16] as a mixture of swapped and un-swapped covalent dimers, indicated as MxM and M = M respectively. The molar ratio of the two isoforms MxM and M = M is 2∶1 at thermal equilibrium. The X-ray structures of the two isomers [17], [18] revealed only small differences in their tertiary structures, located essentially around the 16–22 hinge region, which connects the dislocating N-terminal helix to the protein body. In contrast, there are no differences in the quaternary structures of swapped and unswapped forms, because the inter-subunit interface (the so-called “open-interface” in the 3D domain swapping terminology [2]), pre-exists already in the un-swapped counterpart. This feature reduces significantly the entropic penalty associated with the oligomerization processes. Hence in the case of BS-RNase the dislocation of the N-termini requires only a partial unfolding step associated with a small enthalpic barrier. As a consequence, for this protein the swapping is a physiological, equilibrium process [19] and the molar ratio between swapped and un-swapped forms depends on tiny structural differences that influence the energetic balance. Upon selective reduction of the interchain disulphide bridges, under mild conditions [20], the swapped form of BS-RNase retains a dimeric structure stabilized by non-covalent interactions, whereas the unswapped one is converted into a monomeric derivative, indicated henceforth as mBS.

A comparison of the NMR structure of mBS and RNase A, which share more than 80% of the aminoacid sequence, showed a close similarity between the two proteins except for the 16–22 hinge region, which has higher flexibility in mBS [21]. According to this result and other suggestions, [22], [23], [24], [25], [26], the hinge region may play an important role in the control of the swapping. Inspired by these observations, we have engineered the BS-RNase sequence by substituting either all the different residues of the hinge loop or only the central Pro at position 19, with the corresponding residues of RNase A. Surprisingly, none of these variants displayed significant differences in the hinge region flexibility and swapping extent [19], [27], and the biological activity was only marginally affected [28]. A similar behaviour has been observed also in other swapped proteins, leading to general conclusion that “…based on mutagenesis studies, it is doubtful that sequence features alone determine whether a protein will undergo domain swapping” [2]. On the other hand, 3D domain swapping has been often connected to the instability of the monomeric subunits. Therefore, we decided to perform a more detailed investigation of the structural and dynamical properties of monomeric derivative of BS-RNase. The in depth analysis of the conformational stability of mBS could help to decide if the special features of the swapping dimer arise from an intrinsic property of its subunits, or are rather a consequence of the pre-existence of a dimeric structure. In this paper, we report the refinement of the structure of mBS, together with a characterization of the protein flexibility, chemical stability, surface accessibility and hydration by a combination of NMR techniques and Molecular Dynamics (MD) simulations.

## Results

### Structure refinement

The refined structure of mBS (pdb ID code 2lfj) was calculated out of 2252 unambiguously assigned distance constraints. These correspond to an average of 18 restraints per residue.

**Figure 1:**
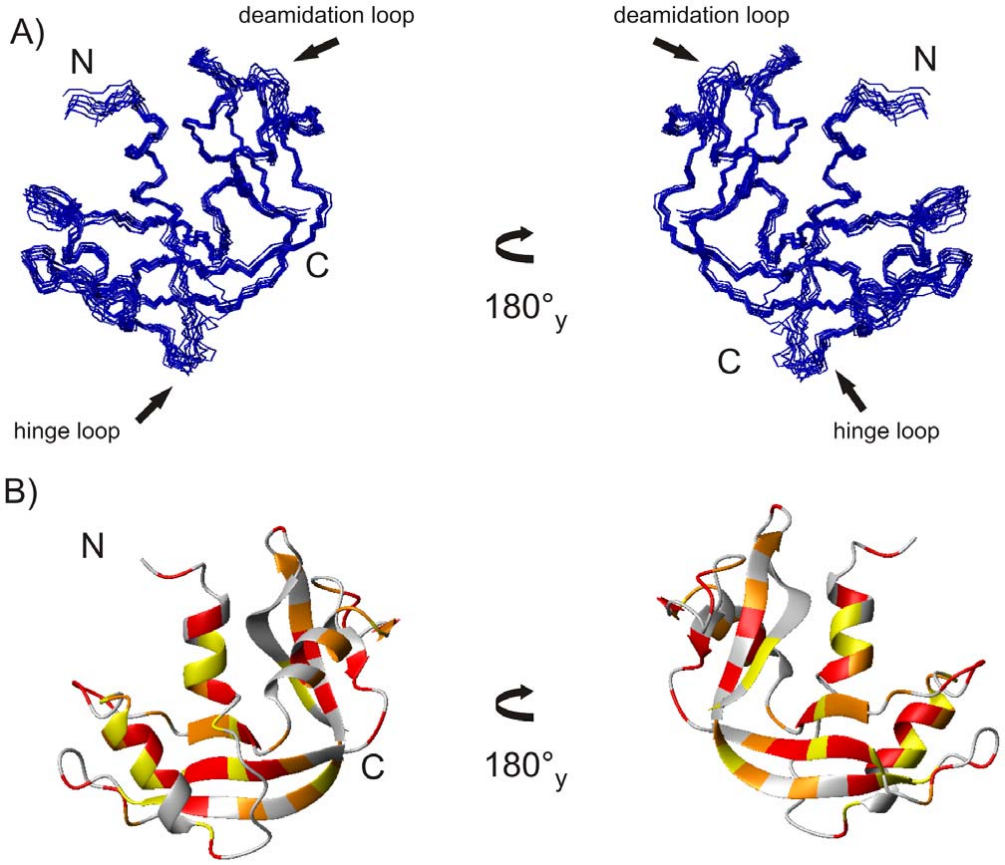
NMR derived structure of mBS. A) Bundle of the best 10 structures of mBS calculated out of 2252 distance constraints (pdb ID code 2lfj); B) protection factors mapped on mBS structure with the following colour code: residues with a P>1×10^5^ in red, the one with 10×10^3^<P<1×10^5^ in orange and the one with P<10×10^3^ in yellow. Residues that couldn't be analyzed are shown in grey.

The resulting structures satisfied the experimental constraints with small deviations from the idealized covalent geometry, 98% of the backbone torsion angles for non-glycine residues being within the allowed regions in the Ramachandram plot. The final structural statistics are reported in Table 1 in comparison with those of the formerly published structure (pdb entry 1QWQ) [21]. The increased number of distance constraints makes the newly refined structures more defined as indicated from the global values of RMSD of the backbone and the side chains, respectively 0.730 and 1.139 Å. A bundle of the 10 structures with lower energies is reported in Figure 1A. In more detail, the higher resolution allowed to extend the third helix by one residue and to observe a better ordered loop spanning residues 65–72, *i.e.* the loop that in the natural enzyme is involved in the deamidation of Asn 67 [29]. Despite the overall higher number of distance constraints, the hinge loop region (residues 16–22) remains among the most flexible regions of the protein, as indicated from the low number of inter-residual NOEs for this region and in agreement with previous [21], [27] and new relaxation data (see below).

**Table 1:** Experimental restraints and structural statistics for ten lowest-energy NMR structures of *mBS-RNase* (2lfj).

	2lfj	1QWQ
**NMR distance constraints**		
Intra-residue, (i−j) = 0	1056	601
Short-range, (i−j) = 1	512	291
Medium-range, 1<(i−j)<5	216	123
Long-range, (i−j)≥5	456	260
Total distance restraints from NOEs	2240	1275
Total distance restraints from S-S bond	12	12
Total distance restraints	2252	1287
NMR dihedral restraints		
Φ, Ψ	104	314
Structural statistics		
RMS deviation for bond angles:	2.0°	1.9°
RMS deviation for bond lengths	0.010 Å	0.010 Å
**Averaged pairwise RMSD (Å)**		
Backbone (global)	0,73±0,12	1.20±0,55
All heavy (global)	1,14±0,14	1.73±0,20
Backbone (secondary structure)^1^	0,45±0,17	0,76±0,10
All heavy (secondary structure)^1^	0,91±0,20	1,32±0,44
Structure quality by procheck analysis^1^		
Residues in most favoured regions	82.8%	72.4%
Residues in additional allowed regions	14.1%	23,9%
Residues in generously allowed regions	1.7%	2,5%
Residues in disallowed regions	1.4%	1,2%

For comparison, also the corresponding data relative to the previous calculated structure (1QWQ) are reported.

1 Residues 4–12,25–31,43–64,70–87,95–112,115–117,122–124.

Regarding the three residues of the catalytic triad, His 119 is present in almost all the structures in one conformation, His 12 is present in two subsets of conformations whereas Lys 41 is very disordered. This result represents a difference with respect to the published crystal structure of the monomer, where the lysine side-chain is bound to a phosphate ion, whereas our solution is deprived of any salt.

### Hydrogen exchange measurements

Further information on mBS stability were derived from hydrogen exchange experiments on ^15^N labeled mBS at pH 5.65, collecting a series of ^1^H-^15^N HSQC experiments at 300 K upon dissolving in D_2_O a lyophilized protein sample.

Exchange constants could be evaluated for 45 well resolved residues distributed all over the sequence, while the H/D exchange process of most of the other amide groups have been only qualitatively determined, see Table 2. Measured exchange constants differ by more than three orders of magnitude along the sequence and were slower by 3 orders of magnitude compared to the intrinsic rates.

**Table 2:** Summary of K_ex_. protection factor (P) and ΔG_op_derived from hydrogen exchange experiments at pH 5.7.

Residue	Kex(hr^−1^)^a^	P	ΔG_op_(kcal mol^−1^)
PHE8	0.114±0.046	8.87E+03	5.40±0.04
GLU9	0.273±0.015	1.82E+03	4.46±0.02
ARG10	0.130±0.018	9.03E+03	5.41±0.06
GLN11	0.026±0.015	9.75E+04	6.82±0.04
HIS12	0.038±0.016	2.48E+05	7.38±0.06
ASP14	0.190±0.027	4.83E+03	5.04±0.01
SER21	0.040±0.100	1.56E+05	7.01±0.03
CYS26	0.109±0.057	4.86E+04	6.41±0.02
LEU28	0.640±0.073	1.15E+03	4.18±0.02
MET30	0.011±0.016	1.53E+05	7.09±0.02
CYS31	0.134±0.019	4.54E+04	6.37±0.01
CYS32	2.220±0.570	6.13E+03	5.18±0.02
ARG33	0.249±0.028	1.85E+04	5.84±0.01
LYS34	0.262±0.017	7.70E+03	5.32±0.05
LYS41	0.200±0.084	1.75E+04	5.80±0.04
VAL43	0.469±0.101	3.39E+02	3.46±0.06
ASN44	0.031±0.010	9.62E+04	6.81±0.09
GLU49	0.044±0.015	6.78E+04	6.61±0.01
ASP53	0.725±0.120	9.90E+02	4.10±0.03
ALA56	0.059±0.006	2.96E+04	6.12±0.02
VAL57	0.003±0.007	7.90E+04	6.70±0.04
CYS58	0.010±0.006	3.39E+05	7.56±0.01
SER59	0.425±0.052	2.12E+04	5.91±0.01
LYS61	0.062±0.075	3.11E+04	6.14±0.02
GLN69	0.026±0.072	8.70E+04	6.76±0.04
GLN74	0.005±0.014	3.43E+05	7.57±0.05
SER77	0.011±0.044	3.73E+05	7.62±0.03
ARG80	0.191±0.026	1.08E+04	5.52±0.02
ILE81	0.007±0.015	5.91E+04	6.53±0.06
THR82	0.005±0.021	1.34E+05	7.01±0.05
ASP83	0.182±0.025	6.19E+03	5.19±0.03
CYS84	0.027±0.017	1.21E+05	6.95±0.04
ARG85	0.016±0.015	2.88E+05	7.47±0.01
GLU86	0.083±0.016	8.63E+03	5.38±0.09
SER89	0.033±0.025	1.40E+05	7.04±0.03
LYS91	0.038±0.013	6.38E+04	6.57±0.10
ALA96	0.621±0.112	6.21E+03	5.19±0.08
TYR97	0.252±0.034	2.84E+03	4.73±0.02
THR100	0.057±0.015	3.15E+04	6.15±0.06
VAL102	0.023±0.010	1.85E+04	5.83±0.02
LYS104	0.014±0.079	5.44E+04	6.48±0.04
ILE106	0.008±0.013	1.61E+05	7.12±0.02
ALA109	0.014±0.014	6.89E+04	6.62±0.07
GLY111	0.322±0.086	2.21E+04	5.94±0.06
VAL118	0.012±0.022	1.30E+04	5.63±0.02
HIS119	0.026±0.012	1.66E+05	7.14±0.05
VAL124	0.360±0.038	3.26E+01	2.07±0.04

a Reported errors refer to the errors in the least square fitting.

For an evaluation of k_op_ protection factors and ΔG based on the exchange rates, it is important to elucidate before the mechanism by which exchange occurs in the conditions employed for the kinetic measurements [30]. It has been reported that under unimolecular exchange regime (EX1) there is no pH dependence of K_ex_, whereas in the bimolecular exchange limit (EX2) K_ex_ will be proportional to the pH [31]. Accordingly, to provide insight into the exchange mechanism we measured the exchange rates also at pH 6.7. The data obtained indicated for BS-RNase an EX2 mechanism, as in the case of RNase A [32].

Measured exchange rates at pH 5.65 were used to calculate protection factors and ΔG_op_, which are reported in Table 2 and plotted in Figure 1B. All the regions of regular secondary structure are well protected from the solvent. As a further evidence of the extreme stability of mBS structure, it's interesting to notice that also residues of some loop regions, such as residues 67–69 belonging to the deamidation loop, exchange slowly.

### Relaxation measurements

**Figure 2:**
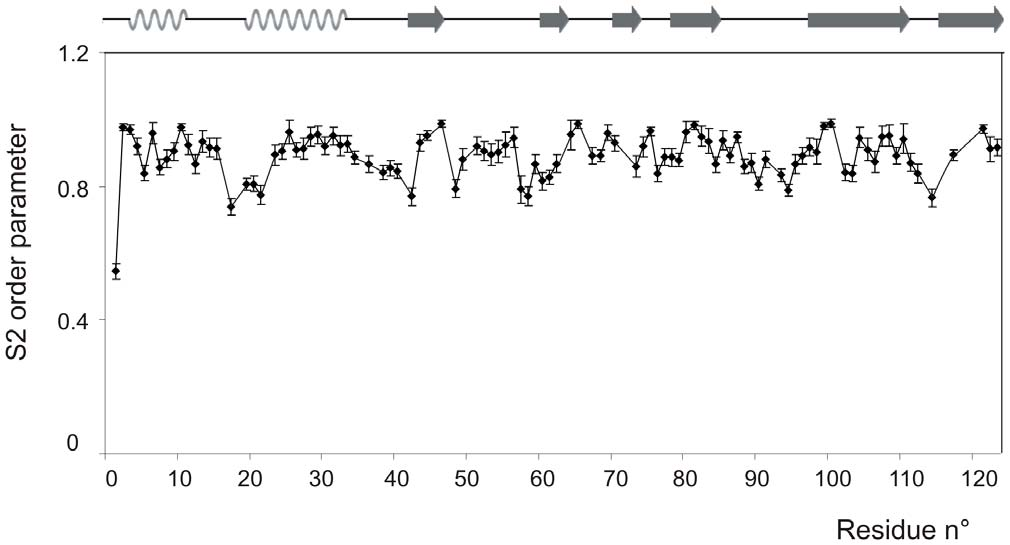
Relaxation data of mBS. S^2^ order parameters of mBS as obtained from the model-free analysis of Lipari and Szabo. (Details are given in the text.) Error bars within the 90% confidence limit were calculated with Montecarlo method Outliers according to R2/R1 rates (residues 70–72) were intentionally omitted from the simultaneous fit of the global correlation time, effective correlation times and order parameters. The mobility of these residues is better demonstrated with the reduced spectral density mapping (see supplementary material).

Conventional ^15^N relaxation data (T_1_,T_2_, NOE), ^15^N/^1^H CSA/DD cross-correlated cross-relaxation rates and heteronuclear NOEs, revealed that residues 2, 18, 20, 21, 22 and 69, 70, 71, 72 were outliers. These data confirm the earlier observations [21], that predicted a high flexibility of the 16–22 hinge and 65–72 loop regions. Residue 2, on the other hand, belongs to the disordered, solvent exposed N-terminal extremity, hence the outstanding values of its relaxation parameters. Independently of the involvement of the R_2_/R_1_ outliers, the model-free evaluation [33] of the T_1_, T_2_ and NOE data set yields τ_c_ = 7.9 ns global correlation time and an average of S^2^ = 0.90±0.07 for the order parameters (overlapping signals and exchange affected residues 70–72 were intentionally omitted from the fit). Similarly to raw relaxation data, S^2^ order parameters also exhibited dips in the hinge region around S18 (Figure 2). The back-calculated ‘theoretical’ T_1_, NOE and T_2_ relaxation data derived from these parameters agreed with the experimental values within 5, 6 and 9% (1 standard deviation), respectively. This modest agreement may be a sign of more mobility, since the typical experimental accuracy of the exponential T_1_ and T_2_ fits was within 2%. Visualisation with reduced spectral density mapping (Figure S1) [34] is a useful complement of the model-free method. On the J(ω_N_)/J(0) spectral density correlation map we see that the bulk of the residues are below the descending part of the limiting curve of the rigid body approach, due to limited internal motions. However, residues that are affected by exchange are right shifted because of increased J(0) contribution (residues 70, 71 and 72). Both approaches gave a dominant monomeric structure, with a correlation time appropriate for this size of proteins. Indeed, a simple empirical formula [35] predicts 8.5 ns correlation time at 300 K for a 124 residue globular protein in water, that is in reasonable agreement with our findings. The small difference might be a consequence of anisotropic rotational diffusion. ^15^N transversal CSA/DD cross-correlated cross-relaxation rates again displays the same mobile regions of mBS (see Figure S1). Combination [36] of ^15^N CSA/DD cross-correlated and conventional relaxation data however, predicted an unlikely high exchange contribution to R_2_ relaxation over the whole sequence. Overall, the relaxation approach described here unequivocally identified two mobile regions in mBS, the hinge 16–22 and the loop 69–72 region, in good agreement with previous heteronuclear NOE data of the same protein and of RNase A. However, careful inspection of the NOE (Figure S2) and S^2^ data anticipates enhanced dynamics of the 113–115 turn region as well. Moreover, extra mobility can persist in the 119–121 region of the β-sheet, as it can be seen from reduced spectral density mapping and R_2_/R_1_ rates (Figure S2). This seems to support the curling of the 115–123 strand in the ensemble of the refined NMR structures.

### Molecular Dynamics simulation

**Figure 3:**
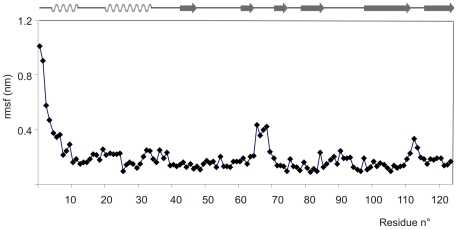
Theoretical Molecular Dynamics of mBS. Root mean square fluctuations (rmsf) values *vs*. mBS sequence.

On the basis of the refined mBS solution structure (pdb ID 2lfj) a 100 ns MD run has been performed in order to predict i) flexibility along the protein backbone, ii) stability of intramolecular hydrogen bonding network and iii) modes of the interaction between mBS and water molecules. In Figure 3 rmsf values are reported for all backbone amides, predicting the highest flexibility for the N terminal moiety. Flexibility is also predicted for 65–68 and 111–113 fragments whose rmsf values are well above the standard deviation, σ = 1.2 Å, calculated for all the residues outside the N terminus. Surprisingly, an average rmsf value of 2.2, just above σ, is observed for the 16–22 hinge loop, that in all the experimental measurements showed a significant flexibility. By analysing the MD trajectory, hydrogen bond percentage lifetimes have been estimated for all mBS backbone amide hydrogens according to previously reported procedures [37], [38]. The obtained results, compared also with the corresponding atom depth indexes, are shown in Table 1S. Reduced involvement in intramolecular hydrogen bonding, as expected, is predicted only for outer backbone amide hydrogens. Density of water molecules in contact with mBS has been derived from the MD trajectory and the distribution of MD hydration sites, MDHS, is also reported in Table 1S.

### Urea unfolding

To characterize the unfolding of mBS we have studied its denaturation in a residue specific manner collecting a series of ^1^H-^15^N HSQC NMR spectra at increasing urea concentration, ranging from 1 to 7 M, and monitoring peak volumes. Figure S3 shows the spectra measured in the presence of 0, 1, 3 and 6 M urea respectively. The HSQC spectra undergo minor chemical shift changes at increasing urea concentration. At low urea concentration (1 M) most of the residues of the monomeric proteins are unaffected from the presence of the denaturant, only the residues of the central strands show a chemical shift perturbation higher than 0.1 ppm. However, by increasing the urea concentration up to 3 M, peaks originating from denatured state appear. As the urea denaturation was found completely reversible, the simultaneous presence of peaks belonging to the folded and unfolded state indicates slow conformational exchange on the NMR chemical shift timescale.

**Figure 4:**
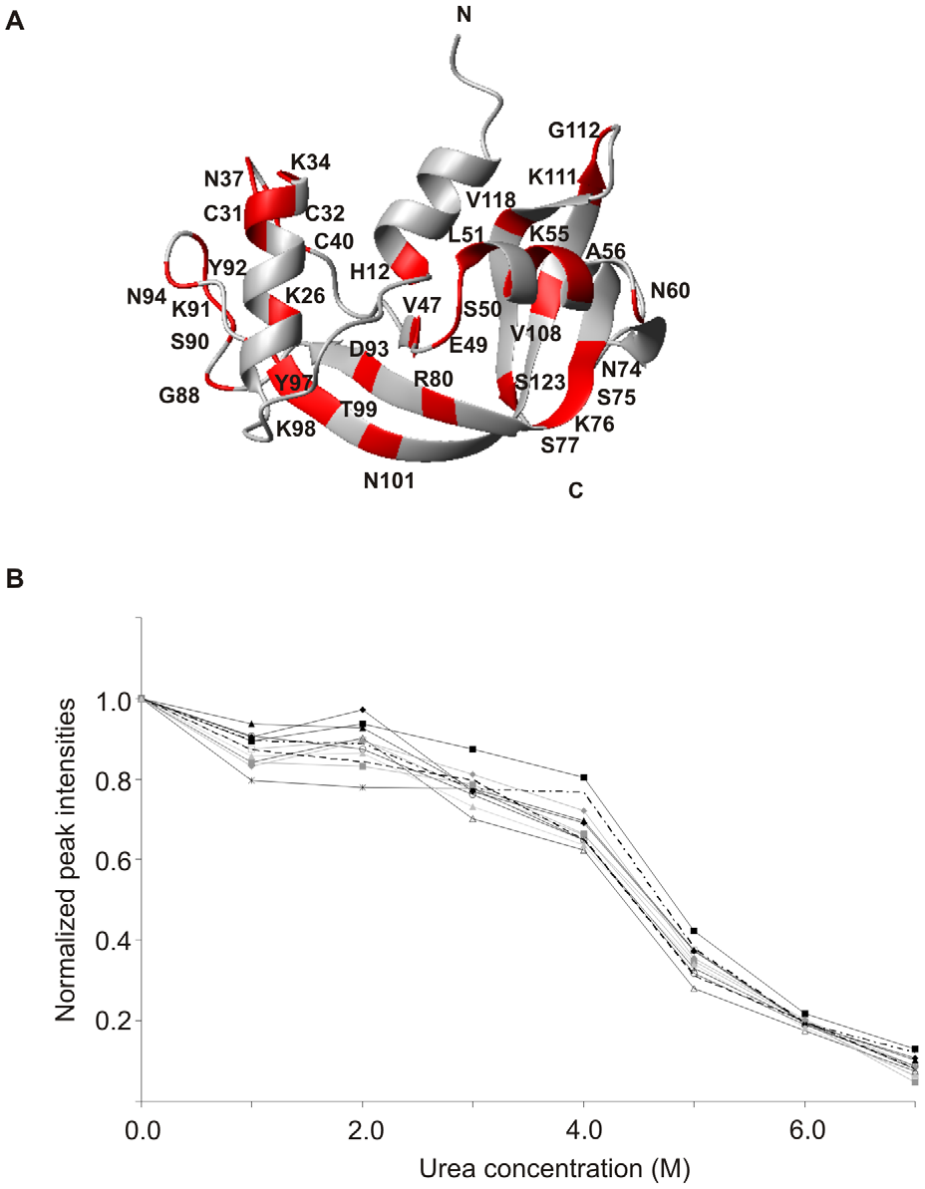
Urea perturbation of mBS structure. A) On mBS structure are shown in red residues whose intensities were still detectable at 6 M urea; B) changes in the normalized NMR peak intensities for selected residues across the range of urea concentration studied. The line/symbol code for each residue is the following:▪ K39, ♦ S77, ▴ L32, ▵ K26, * H12, ▴ G111, dashed line V118, ▪ V54, dashed-dotted line G88, ♦ D83, ○ E49, □- - T36.

The intensities of 30 residues distributed along all the sequence, shown in Figure 4A, could be followed unambiguously up to 6 M urea. This data indicates that still at high urea concentration there is some localized structure. In figure 4B are shown the changes in the normalized NMR peak intensities for selected residues across the range of urea concentrations studied.

A two state mechanism of unfolding, in which only native and denatured molecules are considered [39], fits well with the experimental NMR data. From the data fitting we could calculate the concentration of urea at midpoint of unfolding (C_1/2_). The selected residues show very similar values of C_1/2_, indicating that all the mBS regions behave similarly as a function of urea concentration. The average concentration of urea at midpoint of unfolding is 4.2 M, a value very close to the one obtained monitoring the secondary structure changes at increasing urea concentration by far-UV CD [40]. This result definitely confirms also that the unfolding of mBS follows a two state mechanism, without the presence of intermediate states.

### Paramagnetic profile of TEMPOL accessibility to mBS surface

TEMPOL, the soluble and stable free radical commonly employed for analyzing the distribution of protein surface hot spots [41] has been used as paramagnetic probe for investigating the surface accessibility of mBS. Changes of ^1^H-^15^N signal intensities of backbone amides in HSQC protein spectra, recorded in the presence of variable amounts of TEMPOL in solution, have been measured and reported as paramagnetic attenuations, Ai.

**Figure 5:**
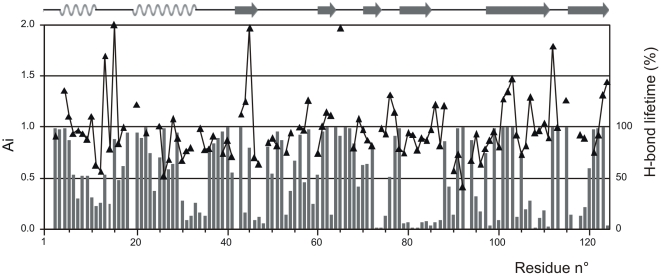
Tempol surface mapping of mBS. Paramagnetic attenuations, Ai, are reported for each well resolved ^1^H-^15^N HSQC signal of mBS (filled triangles). Histogram heights refer to the fractional freedom from intramolecular hydrogen bonding predicted by the 100 ns MD simulation in explicit water (grey bars).

As shown in Table S1 Ai have been calculated for most of mBS amide groups, *i.e.* 87 out of the total 118 well resolved NH signals which are present in diamagnetic and paramagnetic ^1^H–^15^N HSQC spectra. The obtained Ai values range from a maximum of 2.0 to a minimum of 0.4 respectively for signals exhibiting strong and weak paramagnetic attenuations. M13, S15, T45, C65 and G112 residues, exhibiting Ai values higher than 1.7, are the most attenuated amide signals. Interestingly, MD simulation results (*vide supra*) predict that all of them are not tightly involved in intramolecular hydrogen bonding, see Figure 5 and Figure S4, confirming the already suggested bias of TEMPOL to interact with accessible backbone hydrogen bond donors [42].

## Discussion

Although 3D domain swapping was proposed about 50 years ago [3], and it is actually well represented in PDB, its molecular mechanism is still unknown. Eisenberg et al. provided its first definition and proposed a general scheme for this process. In the simplest case “closed” monomeric proteins convert themselves in “open” monomers through a local unfolding step. Then, the interaction between two or more open monomers gives rise to swapped dimers, or even higher oligomers [43]. Very recently, Laurents and coworkers [11] proposed a different mechanism, which involves the transition through an “activated”, unfolded state, re-opening the debate and the scientific interest on the 3D-domain swapping. With respect to the proteins considered by both authors, however, BS-RNase presents still some singularities, which are worth of attention. Indeed, interchange of N-terminal extremities is a physiological, equilibrium process which does not require severe environmental conditions. The two subunits of BS-RNase are covalently linked through two disulfide bonds, so that the two subunits are already correctly oriented for the swapping. *In vivo*, in the reducing cytosol compartment, the un-swapped isomer is supposed to be converted into two monomers, whereas the swapped isoform, stabilized by the interchanged N-terminal helices, retains a dimeric structure [20]. Therefore, BS-RNase is supposed to occur in vivo under multiple forms, depending on the biological compartment encountered, with different tertiary and/or quaternary structures. Given the importance of the 3D domain swapping in the biological properties of BS-RNase, in this paper we have performed a deeper investigation of the structural and dynamical features in solution of its monomeric derivative, with the aim to understand if the BS-RNase subunit has an intrinsic feature to undergo a local unfolding and to be converted into an open monomer, according to the Eisenberg hypothesis [44]. As a first step, we report here the refinement of the solution structure by high resolution NMR spectroscopy up to 0.730 and 1.139 Å resolution for backbone and all atoms respectively, combined with relaxation measurements in solution at 500 MHz and with a characterization of the surface accessibility and hydration at atomic resolution. The increased number of restraints used for structure calculation allowed us a better definition not only of the secondary structure elements, -resulting in a lengthening of helix 3 by one residue- and of the side-chains, but also of the loops, including the 65–72 region. This loop plays a remarkable role in the biological properties of BS-RNase because it includes the Asn-Gly sequence, which spontaneously deamidates under physiological conditions [29], and represents also a potential contact region with Ribonuclease Inhibitor [45], a horse-shoe shaped protein which readily and efficiently inactivates most pancreatic-like ribonucleases. Structural and relaxation data indicate that the mBS behaves like a compact, globular monomer, and the correlation time allowed us to exclude not only the presence of dimeric forms, which instead have been observed in the homologue human pancreatic protein [46], but also a partial opening of the protein. Moreover relaxation data highlight the presence of two main flexible regions, corresponding to the 16–22 and 65–72 loops; in addition, extra-mobility is found at the C-terminal region (112–115 loop and 119–121 strand), *i.e.* in the region involved in the swapping of the C-terminal strand of RNase A [6]. A *cis-trans* isomerisation of proline at position 114 is involved in this process but, despite the presence of a *cis* Pro in the same position, the swapping of the C-terminal ends has never been observed in BS-RNase.

The preferential access of TEMPOL towards few backbone amide groups yields relevant information on mBS surface accessibility. Indeed, the presence of backbone amide groups which are, at the same time, surface exposed, available for hydrogen bonding and TEMPOL accessible, as in the case of M13, S15, C65 and G112 residues, is diagnostic of surface hot spots, belonging to the N-terminal arm (M13, S15), the deamidation loop (C65) and the C-terminal hinge loop (G112). On the other hand, the high paramagnetic attenuation of T45 amide signal is consistent with the expected enhanced accessibility of active site regions [18]. Combined analysis of data obtained from H/D exchange and paramagnetic perturbations indicates that amide groups of residues exhibiting the highest Ai values are also involved in fast H/D isotopic exchange. However, fast isotopic exchange rates do not imply strong paramagnetic perturbations. In this respect, Y92 NH group represents a limiting case, as it exhibits, at the same time, very fast exchange rate and the lowest Ai value. The presence of a MDHS close to Y92 NH could account for local presence of structured water molecules which prevent free TEMPOL approach. It is worth noting that high TEMPOL accessibility of S15 and C65 residues has been already observed in the corresponding positions of the homologous bovine RNase A in a recent multiple solvent crystal structure (MSCS) investigation. In the latter protein, indeed, S15 and C65 residues were in close proximity of R,S,R-bisfuran alcohol and dimethylsulfoxide respectively [47], suggesting some binding properties of these enzyme moieties also in solution.

Thus, high compactness of the protein structure is consistently suggested by the analysis of the H/D exchange data and TEMPOL surface accessibility profiles, as secondary structure elements are all protected from the solvent. This result is very interesting and somehow unexpected, if we consider that helix 1 (residues 4–12) corresponds to the region swapping between the two isoforms, and confirms a close similarity with RNase A structure. In addition, the monitoring of urea denaturation of mBS by NMR allowed us to follow the unfolding process in a residue specific way and to elucidate, eventually, the presence of residues more prone to unfolding and possibly prompting dislocation and swapping. The analysis reported in figure 4 gives us a convincing evidence of a two-step denaturation mechanism for all the protein regions, in agreement with a recent CD and calorimetric study [28], showing that the protein denaturation follows a two step mechanism, and excluding the presence of significantly populated intermediates. On the whole, all data presented in this paper definitely confirm a close similarity with RNase A structure, suggesting also that this protein is extremely compact and there is no evidence of a pre-opening of the monomeric structure in solution. With respect to our previous data [21], this more detailed investigation highlights the presence of potential hot-spots along the protein structure, in particular in the C-terminal region, henceforth we cannot exclude the possibility that, in different and eventually more severe conditions, also the C-terminal swapping could be observed. Regarding the N-terminal hinge region, this is still one of the most flexible regions of the protein but, according to previous mutagenesis studies [19], [48], hinge flexibility together with swapping propensity and *in vitro* antitumor activity [28], are not strictly dependent on a specific sequence.

On the other hand, we cannot exclude other structural and functional roles for the 16–22 loop, which shows a very low similarity with the corresponding region of RNAse A. It is worth to recall here that the presence of a proline replacing Ala 19, in the middle of this region, prevents the subtilisin cleavage [49], and inactivation observed in RNase [50], so that we may hypothesize that the 16–22 hinge sequence of BS-RNase makes the protein more resistant to protease attack. It is also worth to recall here that in a very recent paper by Eisenberg and coworkers [51] the region encompassing Ala 19 in RNase A has been identified as a very good candidate to prompt protein aggregation, thus the possibility that the substitutions present in the hinge region of BS-RNase [52] have been selected to prevent self-assembly phenomena deserves also consideration. For all these reason we believe that the unusual properties of the swapping process of BS-RNase are not strictly dependent on its N-terminal hinge region, as initially supposed, but on the pre-existence of a dimeric structure, independently on the swapping. With respect to RNase A the two extra disulphides at position 31 and 32, that convert the protein into a dimer, on one end facilitate the N-swapping, reducing the entropic disadvantage associated to other 3D domain-swapping cases, all consisting in a momomer/swapped dimer conversion and also preparing the two subunits in the correct orientation, and on the other end create a steric encumbrance which hinders the C-swapping. Finally, the possibility that very subtle structural and dynamical differences induced by inter-subunits interactions increase the protein plasticity should also been considered. Further mutagenesis studies, together with the on-going characterization of the dimers in solution will be helpful to confirm this hypothesis and to understand the molecular basis of the swapping process in BS-RNase.

## Materials and Methods

### NMR experiments

^15^N and ^13^C,^15^N labelled monomeric BS-RNase was expressed and purified as previously described [21]. NMR spectra for structure refinement were acquired at 300 K on Bruker spectrometers operating at 500, 600, 700 and 750 MHz, equipped with triple resonance gradient probes. Data were processed with NMRPipe [53]; visualization of spectra, peak-picking and analysis were done in NMRView [54]. Chemical-shift assignments were obtained from standard three-dimensional triple resonance experiments recorded on ^15^N,^13^C-labeled samples of mBS [55]. NOE-based distance restraints were extracted from a 500-MHz 2D NOESY spectrum, a 500-MHz 3D NOESY-[^1^H,^15^N]-HSQC spectrum, and a 700-MHz 3D NOESY-[^1^H,^13^C]-HSQC spectrum, all with a mixing time of 100 ms. 208 dihedral angle constraints were derived from chemical shifts values using TALOS [56]; additional restraints were obtained from 47 ^3^ *J*_HNHA_ coupling constants extracted from a 500-MHz quantitative *J*-correlated HNHA experiment [57].

The urea induced unfolding of BS-RNase was performed adding solid urea (urea_d4_, Sigma Aldrich) directly into the NMR tube. The urea concentration was increased by 1 M at each titration step until 7 M. In in order to keep protein concentration and pH constant we prepared a different protein sample for each point of the titration and for each of them we adjusted the pH at 5.7. To check for the possible volume increase caused by the addition of solid denaturant, the length of the NMR sample was measured at each step and found constant (within the experimental error). The unfolding process was monitored with a series of 2D HSQC experiments collected at 300 K.

### Structure calculation

Structure calculations were performed by simulated annealing in torsion angle space, using the CYANA 2.0 package [58], which implements an efficient protocol for structure calculation/automated assignment of NOEs. The standard annealing protocol was used with 20000 steps of torsion angle dynamics; in each of seven cycles, 100 structures were calculated, and the 20 with the lowest target function were used in the next stage. In the final run, 200 structures were computed, and the 40 with lowest target function were refined by 4000 steps of restrained minimization in the more realistic AMBER99 force field [59]. To mimic the effect of solvent screening, all net charges were reduced to 20% of their real value, and a distance-dependent dielectric constant (ε = r) was used. The cutoff for non-bonded interactions was 12 Å. The NMR-derived upper bounds were imposed as semi-parabolic penalty functions, with force constants of 16 kcal mol^−1^ Å^−2^; the function was shifted to linearity when the violation exceeded 0.5 Å.

The AMBER-minimized structures were sorted by increasing restraint violation, and the first 10 were selected as the representative bundle. Quality checks were done in PROCHECK-NMR [60], while MOLMOL [61] was used for structure visualization and analysis.

### Molecular Dynamics simulations

MD simulations were performed in explicit solvent starting from the lowest energy NMR structure of (pdb ID 2lfj). GROMACS package [62] and the AMBER force field [63] were used for the 100 ns MD run of solvated structure in a cubic box of equilibrated TIP3P water molecules [64]. The initial shortest distance between the protein and the box boundaries was set to 1.0 nm and chloride ions were added to achieve global electric neutrality. Afterwards, the system was energy minimized with 900 steps of conjugate gradients. To achieve a good equilibration prior to the long MD simulations, the system was subjected to a short (20.0 ps) MD run where the atoms of the macromolecule were restrained to their positions, allowing only the solvent to move. The protein-water system was simulated in the NPT ensemble by keeping constant the temperature (300 K) and pressure (1 atm); a weak coupling to external heat and pressure baths was applied (relaxation times were 0.1 ps and 0.5 ps, respectively). Bonds were constrained by LINCS algorithm, while non-bonded interactions were accounted by using the PME method (grid spacing 0.12 nm) [65] for electrostatic contribution and 0.9 nm cut-off for VDW contribution. An integration time step of 2 fs was used and trajectory snapshots were saved every 0.2 ps. As the backbone RMSD levels off after an equilibration period of 100 ps subsequent analysis of MD trajectories was carried out from 100 ps onward. Cut-off values of 0.35 nm and 75° between donor and acceptor moieties have been considered for hydrogen bonding. The fraction of the MD trajectory in which hydrogen bond criteria are fulfilled for a given donor is denoted by “hydrogen bond percentage life time” [37], [38]. Solvent density map, whose maxima have been defined as molecular dynamics hydration sites (MDHS's) [66], [67], were calculated from the atomic coordinates of the explicit waters in the simulation. The space surrounding the protein was divided into two shells: the first one extended up to a distance of 0.6 nm from the protein surface and accounted for the protein hydration sites; the second one, representing the bulk solvent, extended from 0.6 nm to 0.8 nm from the protein surface. The water positions were accounted in a 3D grid (step-size 0.05 nm). For each frame, the protein was superimposed onto a reference structure in order to eliminate the effects of translations and rotations. 3D iso-contour plot of the resulting water density was obtained by using PyMOL software (http://www.pymol.org).

### Hydrogen exchange

Hydrogen exchange experiments were performed dissolving the ^15^N-labelled lyophilised NMR protein sample in 99.9% D_2_O. A series of successive 2D ^1^H-^15^N HSQC spectra was recorded and 300 K. Fifty hydrogen exchange rates were determined by fitting a first order exponential equation to the peak heights versus time data:
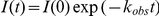
where I (t) represents the cross peak height at time t, I(0) the original cross peak height, k_obs_ the observed rate of hydrogen exchange and t the time in seconds. The intrinsic hydrogen exchange rates k_rc_ were calculated using the web server program Sphere (http://www.fccc.edu/research/labs/roder/sphere, Yu-Zhu Zhang, Protein and peptide structure and interactions studied by hydrogen exchange and NMR. *Ph.D. Thesis*, Structural Biology and Molecular Biophysics, University of Pennsylvania, PA, USA.). The protection factor (PF) for each well resolved NH group was then calculated using the equation:
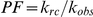
The free energy of opening, ΔG_op_, was calculated from the equation
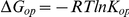
where k_op_ = k_obs_/k_rc_. Data were fitted with the program KaleidaGraph 3.51 (Synergy software) [68].

### Relaxation

For NMR dynamics, besides the conventional ^15^N relaxation (T_1_, T_2_ and ^15^N-NOE) [69], the longitudinal and transverse ^15^N and ^1^H CSA/DD cross-correlated relaxation rates η_zz_(^15^N), η_xy_(^15^N), η_xy_(^1^H) [46], were measured at 500.13 MHz on a Bruker DRX spectrometer. To this end, a series of 2D ^1^H-^15^N HSQC spectra using sensitivity enhanced gradient pulse schemes [70] were recorded. The typical experimental parameters are summarized as follows. The ^1^H carrier frequency was set to the water resonance at 4.7 ppm using 15 ppm window, while the ^15^N window was 28 ppm centred at 118.5 ppm. The relaxation delay times were set as follows for T_1_: 11.2, 101.2, 201.2, 401.2, 601.2, 801.2, 1001.2, 1201.2 ms; for T_2_, CPMG pulse trains of 0.03, 30.4, 60.8, 91.2, 121.6, 182.4, 243.2, 302.4, 360, 417.6 ms in duration were used; for the measurement of cross-correlated relaxation rates, η_zz_ and η_xy_, the relaxation interference was allowed to be active for 20 or 21.6 ms in pairs of experiments including the reference (Δ = 0 ms) experiment. The number of transients collected per t_1_ increment was 8 for T_1_, 16 for T_2_, 32 for NOE, 128 for η_zz_ and 80 for η_xy_ measurements. A spin-lock field of 3400 Hz was used for the ^15^N transverse cross-correlation experiment. Two-parameter exponential fits of the measured volume intensities of cross-peaks were applied to extract the relaxation times T_1_ and T_2_. The cross-correlation rate constants were determined using the initial linear build-up rate approach. The theoretical expressions for the autorelaxation (R_1_, R_2_) and cross-correlation rate constants (η_xy_, η_zz_) and for the steady-state heteronuclear NOE in terms of the spectral density functions (J^a^(ω) auto- and J^c^(ω) cross-correlation) are used as given in the literature [71]. The simplifying assumption of isotropic overall tumbling and the axial symmetry of constant (Δσ = −160 ppm) ^15^N chemical shielding tensors were applied. A bond length of *r_NH_* = 0.102 nm was used in all calculations. The model-free [33] analysis of T_1_, T_2_ and heteronuclear NOE yielded the S^2^ order parameters and local correlation times for most amides and the global correlation time.

### Paramagnetic studies of Tempol induced perturbations

^1^H–^15^N HSQC diamagnetic and paramagnetic spectra, recorded from a Bruker DRX 600 spectrometer with 512 increments and 128 scans over 2048 data points, were compared to determine paramagnetic perturbations on signal intensities.

Only well resolved NMR signals have been considered for quantifying cross-peak volumes in the presence and in the absence of the paramagnetic probes, respectively *V_i_^d^* and *V_i_^p^*. Such volumes have been measured with an estimated error lower than 10% and their autoscaled values, υ_i_, were used according to the relation:
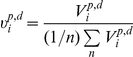
Paramagnetic attenuations, A_i_, were calculated from the autoscaled diamagnetic and paramagnetic peak volumes, respectively, υ_d_ and υ_p_, according to the relation:
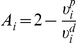



### Atom depth calculations

By using the SADIC suite [72], atom depth of all the hydrogen of mBS backbone amide groups have been calculated. A radius of 8 Å for the probing sphere was employed to measure exposed volumes and, hence, depth indexes, Di.

## Supporting Information

Table S1 **Experimental and calculated parameters obtained for solvent-protein interactions.**
(DOC)

Figure S1 **Relaxation data of mBS.** A) Residue by residue representation of ^15^N spectral densities as a function of low-frequency components. The continuous curve represent a rigid body, with 8 ns global correlation time. The outliers to the right of the curve clearly identify residues 70–72 as the most exchange affected part of mBS; B) Transversal ^15^N-^1^H CSA-DD cross-correlated cross-relaxation rates of the amides in mBS. Lower rates can be attributed higher internal mobility.
(TIF)

Figure S2 **Relaxation measurments on mBS.** A) Heteronuclear ^15^N-{^1^H} NOE values in mBS; B) Ratio of ^15^N T_1_/T_2_ relaxation times in mBS.
(TIF)

Figure S3 **Urea denaturation experiments.** ^1^H-^15^N HSQC spectra of mBS at different urea concentrations.
(TIF)

Figure S4 **Surface accessibility of mBS.** Correlation diagram between TEMPOL-induced paramagnetic perturbations and fractional HB freedom predicted by MD simulation. The least squares linear fit, represented by the straight line, yielded the following parameters: slope 0.22, intercept 0.81, correlation coefficient 0.18.
(TIF)
