# Correction: NMR Studies on Structure and Dynamics of the Monomeric Derivative of BS-RNase: New Insights for 3D Domain Swapping

**DOI:** 10.1371/annotation/b2acf16f-53da-4a8f-a46f-573b7f6f08b7

**Published:** 2012-02-28

**Authors:** Roberta Spadaccini, Carmine Ercole, Maria A. Gentile, Domenico Sanfelice, Rolf Boelens, Rainer Wechselberger, Gyula Batta, Andrea Bernini, Neri Niccolai, Delia Picone

There was an error in the funding statement. The correct funding statement is: "This work was supported by grants OTKA (Hungarian Scientific Research Fund) CK-77515 and TAMOP (Operative Program for the Renewal of the Society) 4.2.1/B-09/1/KONV-2010-0007 (GB) (Convergence) and 20088L3BP3_001 (DP). EU-NMR and the Utrecht Large Scale Facility are acknowledged for support. Financial support for access to EAST-NMR Research Infrastructures in Debrecen (7th Framework Programme of the EC, Contract 228461) is also gratefully acknowledged. CE was recipient of a San Paolo BN fellowship (Progetto FORGIARE). The funders had no role in study design, data collection and analysis, decision to publish, or preparation of the manuscript." 

