# Supplementary material for: NMR Studies on Structure and Dynamics of the Monomeric Derivative of BS-RNase: New Insights for 3D Domain Swapping
Source: PLoS One. 2012 Jan 12;7(1):e29076. doi: 10.1371/journal.pone.0029076 (PMC3257227; doi:10.1371/journal.pone.0029076)
Supplement: Table S1 — Experimental and calculated parameters obtained for solvent-protein interactions. (DOC) [file pone.0029076.s001.doc]

**Table S1.**Experimental and calculated parameters obtained for solvent-protein interactions

| **Residue** | **ESA (A^2^)^b^** | **A_i_^c^** | **H/D exchange^d^** | **ss^e^** | **MDHS^f^** |
| --- | --- | --- | --- | --- | --- |
| LYS1 | 34.9 | n/a | n/a | c | n |
| GLU2 | 4.4 | 0.91 | very slow | c | n |
| SER3 | 3.7 | n/a | n/a | c | n |
| ALA4 | 3.9 | 1.36 | fast | h | n |
| ALA5 | 2.6 | 1.11 | n/a | h | n |
| ALA6 | 1.5 | 0.94 | very slow | h | n |
| LYS7 | 0.6 | 0.97 | n/a | h | n |
| PHE8 | 0.9 | 0.94 | slow | h | n |
| GLU9 | 1.2 | 0.88 | slow | h | n |
| ARG10 | 0.3 | 1.11 | slow | h | n |
| GLN11 | 0.1 | 0.63 | slow | h | n |
| HIS12 | 0.1 | 0.57 | slow | h | y |
| MET13 | 0.6 | 1.70 | fast | c | y |
| ASP14 | 0.1 | 0.78 | slow | c | n |
| SER15 | 1.0 | 2.00 | fast | c | y |
| GLY16 | 1.4 | 0.83 | fast | c | n |
| ASN17 | 1.4 | 0.99 | fast | c | y |
| SER18 | 3.3 | n/a | fast | c | n |
| SER20 | 2.4 | 1.22 | fast | c | n |
| SER21 | 1.3 | n/a | slow | h | n |
| SER22 | 5.2 | 0.94 | intermediate | h | y |
| SER23 | 1.3 | n/a | fast | h | y |
| ASN24 | 0.2 | n/a | n/a | h | y |
| TYR25 | 1.3 | 1.01 | fast | h | n |
| CYS26 | 1.8 | 0.52 | slow | h | n |
| ASN27 | 0.9 | 0.68 | intermediate | h | y |
| LEU28 | 1.4 | 1.08 | slow | h | n |
| MET29 | 0.8 | 0.89 | very slow | h | y |
| MET30 | 0.0 | 0.67 | slow | h | n |
| CYS31 | 0.0 | 0.77 | slow | h | n |
| CYS32 | 0.2 | 0.79 | slow | h | n |
| ARG33 | 0.0 | n/a | slow | h | n |
| LYS34 | 0.1 | 0.99 | slow | h | n |
| MET35 | 0.1 | 0.78 | n/a | c | n |
| THR36 | 0.8 | 0.79 | fast | c | n |
| GLN37 | 2.2 | 0.91 | very slow | c | n |
| GLY38 | 2.5 | n/a | fast | c | n |
| LYS39 | 2.0 | 0.74 | very slow | c | n |
| CYS40 | 2.8 | 0.87 | fast | c | n |
| LYS41 | 0.1 | 0.71 | slow | c | y |
| VAL43 | 0.7 | 1.13 | slow | s | n |
| ASN44 | 0.4 | 1.25 | n/a | s | y |
| THR45 | 1.9 | 1.97 | intermediate | s | y |
| PHE46 | 0.0 | 0.70 | very slow | s | n |
| VAL47 | 0.2 | 0.64 | very slow | s | n |
| HIS48 | 0.0 | n/a | n/a | c | n |
| GLU49 | 0.1 | 0.84 | slow | c | n |
| SER50 | 2.3 | 0.89 | fast | h | y |
| LEU51 | 4.0 | 0.81 | n/a | h | y |
| ALA52 | 2.6 | n/a | n/a | h | n |
| ASP53 | 0.1 | 0.75 | slow | h | n |
| VAL54 | 0.0 | 0.95 | very slow | h | n |
| LYS55 | 0.3 | n/a | n/a | h | n |
| ALA56 | 1.9 | 1.00 | slow | h | n |
| VAL57 | 0.7 | 0.97 | slow | h | y |
| CYS58 | 1.5 | 1.26 | slow | h | y |
| SER59 | 0.1 | n/a | slow | h | n |
| GLN60 | 0.7 | 0.74 | intermediate | c | n |
| LYS61 | 1.6 | 1.01 | slow | s | y |
| LYS62 | 5.4 | 1.14 | very slow | s | n |
| VAL63 | 0.1 | 1.11 | n/a | s | n |
| THR64 | 4.1 | n/a | fast | s | y |
| CYS65 | 4.0 | 1.97 | fast | c | n |
| LYS66 | 3.9 | n/a | fast | c | n |
| ASP67 | 2.4 | n/a | very slow | c | n |
| GLY68 | 3.1 | 0.80 | very slow | c | n |
| GLN69 | 1.6 | 1.08 | slow | c | n |
| THR70 | 0.8 | 0.98 | fast | c | n |
| ASN71 | 2.3 | 0.87 | fast | s | n |
| CYS72 | 1.8 | 0.81 | fast | s | n |
| TYR73 | 0.1 | n/a | n/a | s | n |
| GLN74 | 0.0 | 0.98 | slow | s | y |
| SER75 | 0.3 | 0.93 | very slow | c | y |
| LYS76 | 1.8 | 1.32 | fast | c | n |
| SER77 | 2.0 | 1.14 | slow | c | n |
| THR78 | 3.4 | 0.79 | fast | c | y |
| MET79 | 0.0 | 0.75 | n/a | s | n |
| ARG80 | 0.2 | 0.94 | slow | s | n |
| ILE81 | 0.2 | 0.93 | slow | s | y |
| THR82 | 0.1 | 0.77 | slow | s | n |
| ASP83 | 0.3 | 0.89 | slow | s | n |
| CYS84 | 0.0 | 0.87 | slow | s | n |
| ARG85 | 0.0 | 0.97 | slow | s | n |
| GLU86 | 0.2 | 1.22 | slow | c | y |
| THR87 | 0.0 | 0.82 | fast | c | n |
| GLY88 | 0.1 | 1.21 | fast | c | y |
| SER89 | 0.6 | n/a | slow | c | n |
| SER90 | 0.5 | 0.58 | very slow | c | n |
| LYS91 | 3.2 | 0.73 | slow | c | n |
| TYR92 | 3.4 | 0.41 | fast | c | y |
| ASN94 | 0.4 | 0.67 | very slow | c | n |
| CYS95 | 0.9 | 0.93 | fast | c | y |
| ALA96 | 0.1 | 0.64 | slow | c | n |
| TYR97 | 3.6 | 0.79 | slow | c | n |
| LYS98 | 0.0 | 0.87 | very slow | s | n |
| THR99 | 4.3 | 0.95 | fast | s | y |
| THR100 | 0.1 | 0.80 | slow | s | y |
| GLN101 | 4.2 | 1.27 | fast | s | y |
| VAL102 | 0.3 | 1.35 | slow | s | n |
| GLU103 | 4.4 | 1.47 | intermediate | s | y |
| LYS104 | 0.0 | 0.92 | slow | s | n |
| HIS105 | 1.2 | 0.73 | fast | s | y |
| ILE106 | 0.3 | 0.82 | slow | s | n |
| ILE107 | 0.1 | 1.29 | n/a | s | n |
| VAL108 | 0.0 | 0.94 | very slow | s | n |
| ALA109 | 0.2 | 0.96 | slow | s | n |
| CYS110 | 0.7 | 1.04 | n/a | s | n |
| GLY111 | 0.1 | 0.89 | slow | s | n |
| GLY112 | 8.0 | 1.79 | fast | c | n |
| LYS113 | 5.9 | 0.99 | very slow | c | n |
| PRO114 | 0.0 | n/a | n/a | c | n |
| SER115 | 5.7 | 1.26 | n/a | c | n |
| VAL116 | 0.0 | n/a | n/a | s | n |
| PRO117 | 0.0 | n/a | n/a | s | n |
| VAL118 | 0.0 | 0.92 | slow | s | n |
| HIS119 | 0.1 | 0.89 | slow | s | n |
| PHE120 | 1.9 | n/a | n/a | s | n |
| ASP121 | 2.7 | 0.75 | intermediate | s | y |
| ALA122 | 1.5 | 0.92 | intermediate | s | y |
| SER123 | 3.8 | 1.31 | fast | s | y |
| VAL124 | 0.0 | 1.44 | slow | s | n |
| For all non prolyl residues of mBS the following parameters are give: a) ffHB is the fractional freedom from intramolecular hydrogen bonding calculated for each protein backbone amide; b) averaged exposed surface areas calculated during the MD trajectory; c) auto-scaled paramagnetic attenuations of ^15^N-^1^H HSQC signals; d) hydrogen deuterium exchange rates grouped in four different classes (see text); e) presence in secondary structure elements (c = coil, h = helix and s = strand); f) occurrence of MD hydration sites with maxima located within 0.6 nm from amide nitrogen atoms (**y**es or **n**ot). | | | | | |
