# Supplementary figures and images for: NMR Studies on Structure and Dynamics of the Monomeric Derivative of BS-RNase: New Insights for 3D Domain Swapping
Source: PLoS One. 2012 Jan 12;7(1):e29076. doi: 10.1371/journal.pone.0029076 (PMC3257227; doi:10.1371/journal.pone.0029076)

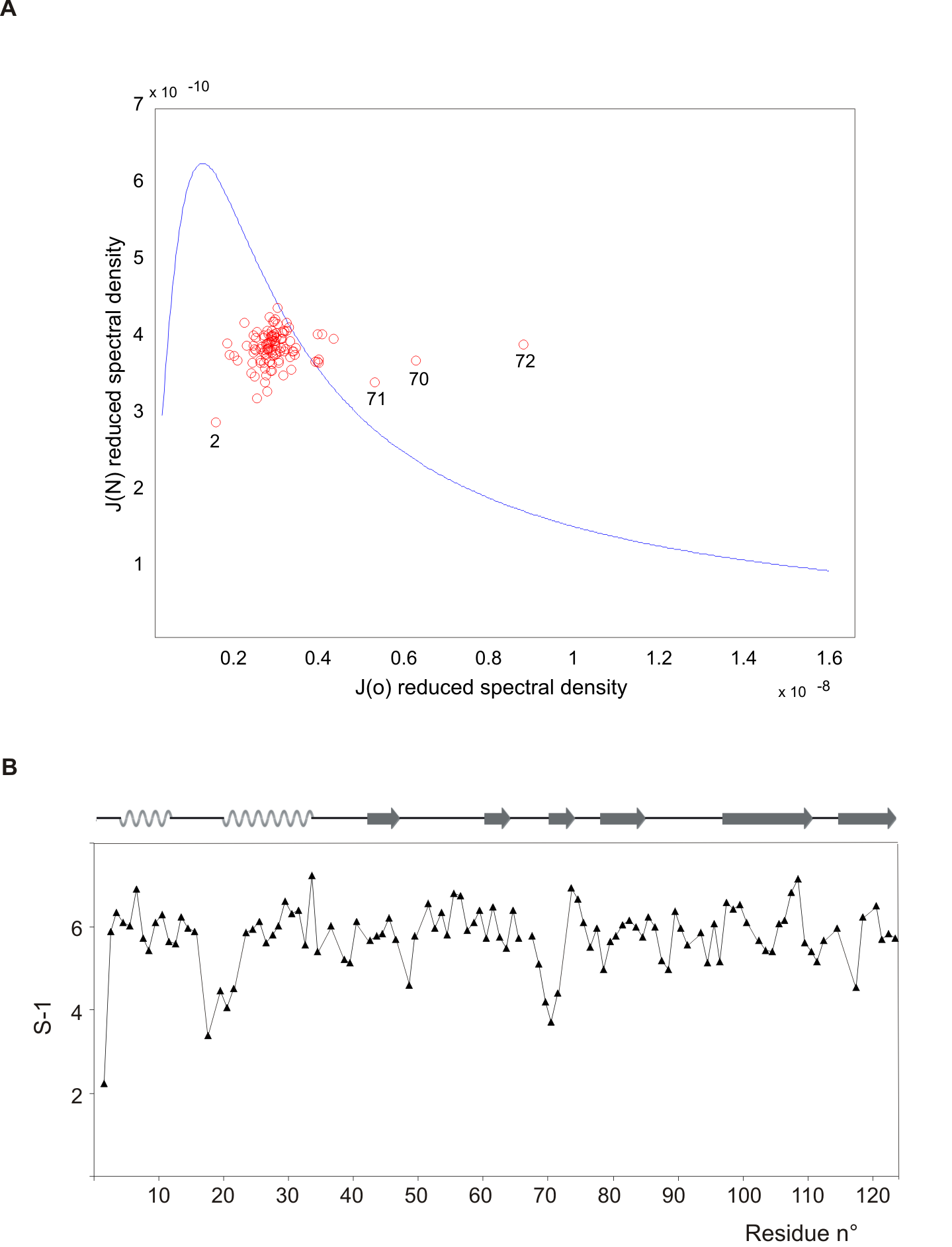

Supplement: Figure S1 — Relaxation data of mBS. A) Residue by residue representation of 15N spectral densities as a function of low-frequency components. The continuous curve represent a rigid body, with 8 ns global correlation time. The outliers to the right of the curve clearly identify residues 70–72 as the most exchange affected part of mBS; B) Transversal 15N-1H CSA-DD cross-correlated cross-relaxation rates of the amides in mBS. Lower rates can be attributed higher internal mobility. (TIF) [file pone.0029076.s002.tif]

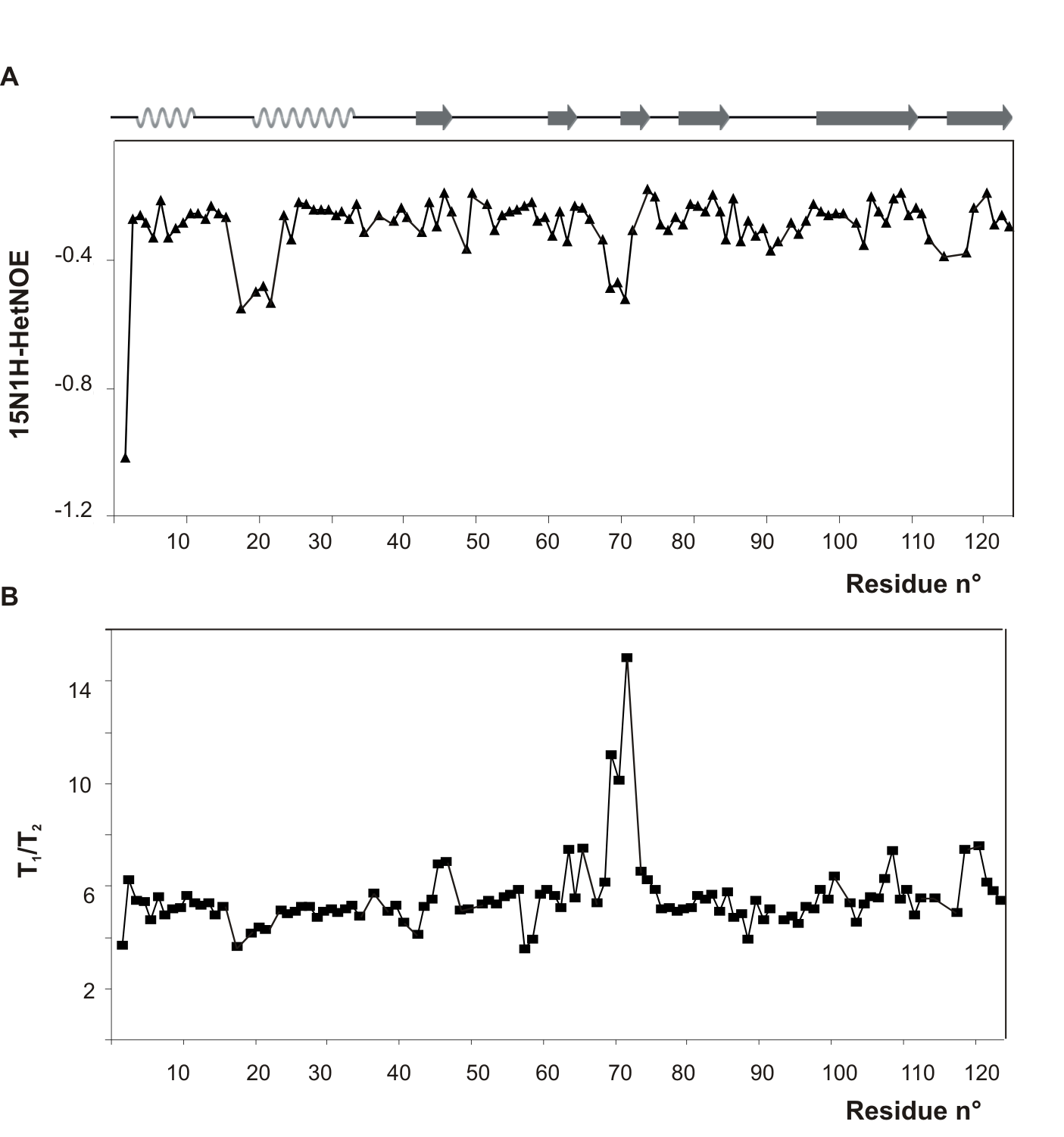

Supplement: Figure S2 — Relaxation measurments on mBS. A) Heteronuclear 15N-{1H} NOE values in mBS; B) Ratio of 15N T1/T2 relaxation times in mBS. (TIF) [file pone.0029076.s003.tif]

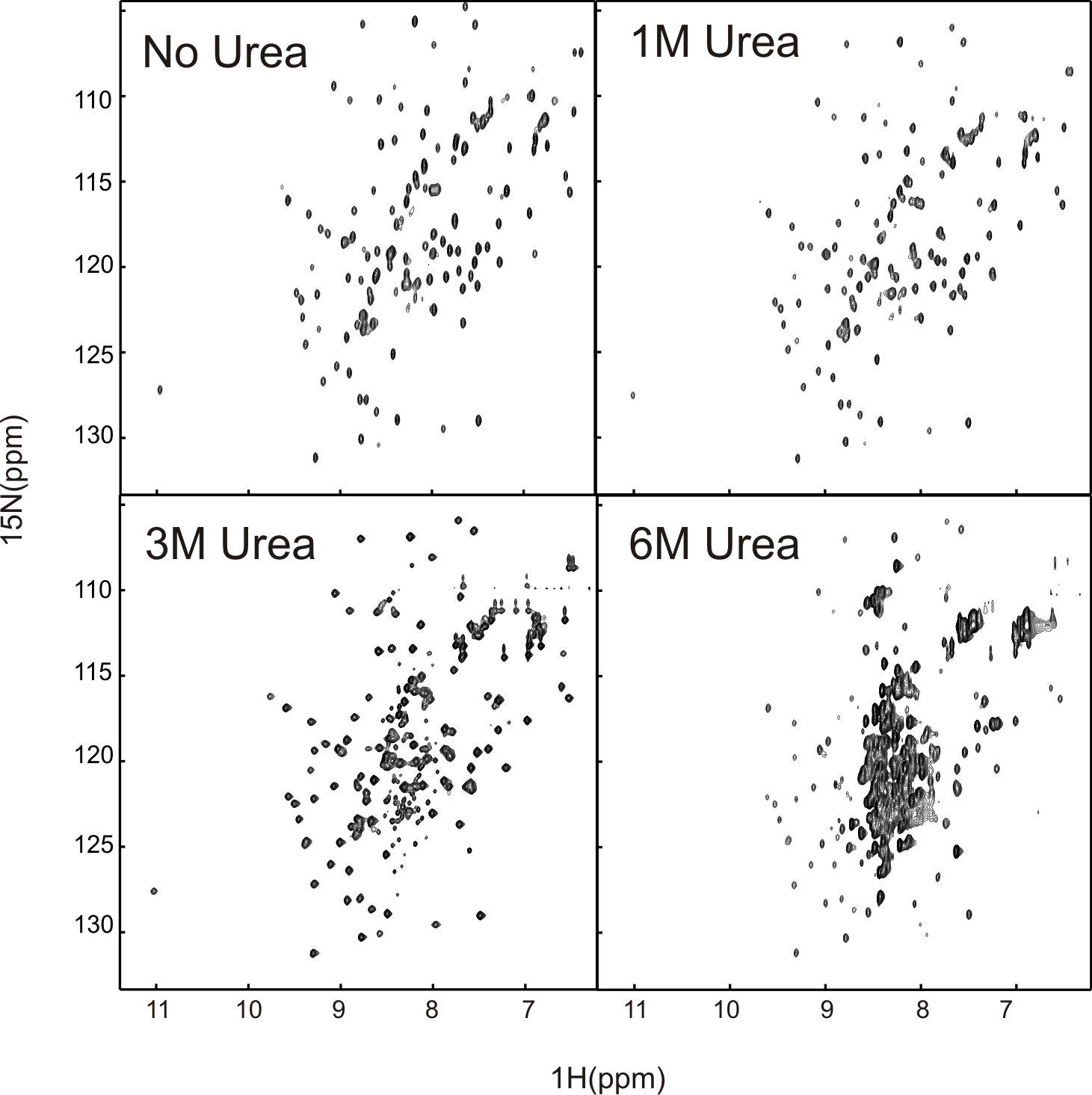

Supplement: Figure S3 — Urea denaturation experiments. 1H-15N HSQC spectra of mBS at different urea concentrations. (TIF) [file pone.0029076.s004.tif]

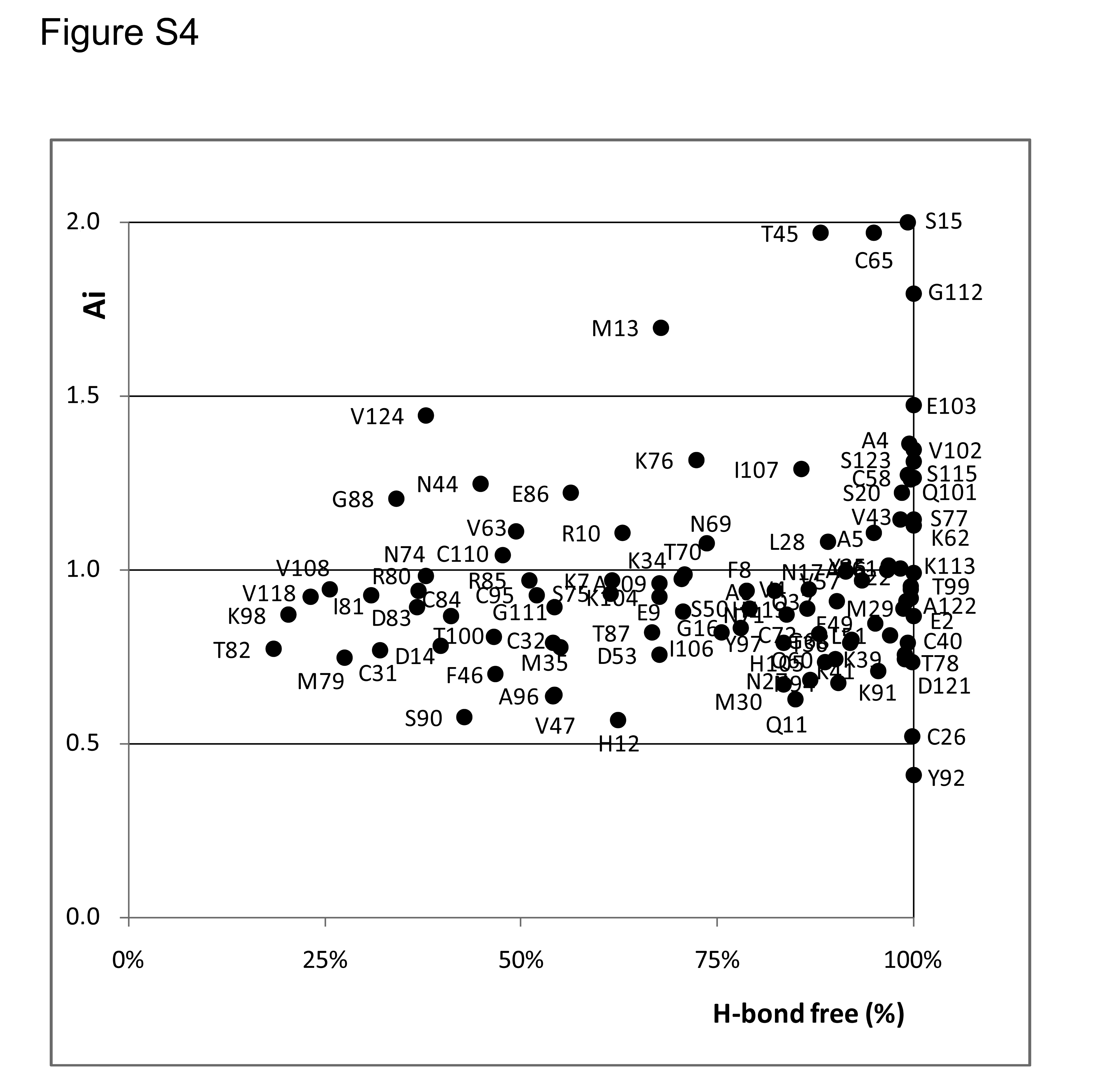

Supplement: Figure S4 — Surface accessibility of mBS. Correlation diagram between TEMPOL-induced paramagnetic perturbations and fractional HB freedom predicted by MD simulation. The least squares linear fit, represented by the straight line, yielded the following parameters: slope 0.22, intercept 0.81, correlation coefficient 0.18. (TIF) [file pone.0029076.s005.tif]
